# miR-9: a versatile regulator of neurogenesis

**DOI:** 10.3389/fncel.2013.00220

**Published:** 2013-11-20

**Authors:** Marion Coolen, Shauna Katz, Laure Bally-Cuif

**Affiliations:** Zebrafish Neurogenetics Team, Laboratory of Neurobiology and Development, Institute of Neurobiology Alfred Fessard, CNRSGif-sur-Yvette, France

**Keywords:** microRNA-9, neurogenesis, embryonic progenitors, neural stem cells, proliferation

## Abstract

Soon after its discovery, microRNA-9 (miR-9) attracted the attention of neurobiologists, since it is one of the most highly expressed microRNAs in the developing and adult vertebrate brain. Functional analyses in different vertebrate species have revealed a prominent role of this microRNA in balancing proliferation in embryonic neural progenitor populations. Key transcriptional regulators such as FoxG1, Hes1 or Tlx, were identified as direct targets of miR-9, placing it at the core of the gene network controlling the progenitor state. Recent data also suggest that this function could extend to adult neural stem cells. Other studies point to a role of miR-9 in differentiated neurons. Moreover miR-9 has been implicated in human brain pathologies, either displaying a protective role, such as in Progeria, or participating in disease progression in brain cancers. Altogether functional studies highlight a prominent feature of this highly conserved microRNA, its functional versatility, both along its evolutionary history and across cellular contexts.

microRNAs are small regulatory RNAs that modulate, generally negatively, the translation and/or stability of mRNA targets through complementary binding to their 3' untranslated region (3'UTR; Bartel, 2009). microRNA genes are transcribed as primary transcripts (pri-miR), which are cleaved in the nucleus to generate precursor transcripts (pre-miR) (Yang and Lai, 2011). One characteristic of pre-miR is that they form a secondary hairpin structure. Pre-miR are exported to the cytoplasm where they are further cleaved by the enzyme Dicer. This cleavage gives rise to a duplex of short RNA strands (the 5' and 3' strands), one of which is then loaded into the RNA-induced silencing complex (RISC). The discovery of microRNAs unraveled a new layer of complexity of gene regulatory networks. Computational and experimental approaches have demonstrated that a single microRNA can regulate the expression of hundreds of mRNA targets. However, despite their large spectrum of action, loss of microRNA function often results in paradoxically subtle phenotypes, some of which are only apparent in a sensitized genomic or environmental context (Li et al., 2009). In the light of these findings, microRNAs are hypothesized to confer robustness to developmental programs, and to facilitate transitions between different cellular states (Takacs and Giraldez, 2010; Ebert and Sharp, 2012). Therefore, studying their implication during neurogenesis, which implies the transition of a neural progenitor to a differentiated neuronal cell, might shed new light on regulation of this process. microRNA-9 (miR-9) captured the attention of neurobiologists because of its high sequence conservation in bilaterian animals, and its high and specific expression in the central nervous system (CNS) of vertebrates. As its function and spectrum of action begins to be unraveled, this microRNA proves to be highly versatile, exerting various and sometimes opposite activities depending on the species and cellular context.

## Evolutionary history of *miR-9* genes

### Structural evolution of the *miR-9* gene family

**Figure 1:**
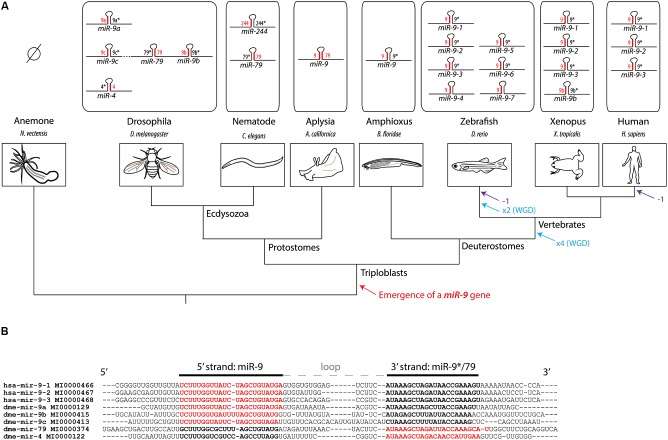
**History of the miR-9 gene family.** **(A)** Phylogenetic tree showing the evolutionary relationships between different model species and the composition of the *miR-9* gene family in their respective genomes. The preferred microRNA strand is represented in red, while the non-preferred (or “star”) is represented in black. No *miR-9* gene has been recovered so far from genomes of cnidarian species, such as the sea anemone or hydra, suggesting that the emergence of a *miR-9* gene occurred at the transition towards triploblasty. At the origin of jawed vertebrates, two rounds of whole genome duplications (WGD) have occurred. An additional WGD occurred in the teleost lineage. These duplication events likely account for the presence of multiple miR-9 genes in vertebrates. Eutherian mammals lost one class of *miR-9* genes (corresponding to *miR-9*-4, also called *miR-9b*). **(B)** Alignment of pre-miR-9 sequences from *Drosophila* and human. Sequences, names and reference numbers were retrieved from miRBase.^1^ Nucleotides highlighted in bold correspond to the two microRNA strands, with the preferred strand in red, and the non-preferred one in black.

The *miR-9* gene is ancient in animal evolution, as it appeared at the transition towards triploblasty (Wheeler et al., 2009). The genome of some extant animal species contains several copies of this gene (Figure 1A). In Vertebrates, the amplification of *miR-9* genes parallels the whole genome duplication events that occurred in the phylum and thus likely results from them. Independent duplications events also occurred in other phyla such as arthropods. This led in particular to the presence of five *miR-9* genes in *Drosophila*, three of which are clustered in the same gene complex (Lai et al., 2004). The level of sequence conservation of pre-miR-9 among animals is strikingly high, in particular at the level of both 5' and 3' mature microRNA strands. For instance the 5' strand of drosophila *miR-9*a gene is identical to the one in human genes (Figure 1B). The retention in vertebrate genomes of multiple independent copies of a gene generating identical microRNA forms is quite surprising. It could be linked to differential expression of paralogous *miR-9* genes, leading to subfunctionalization between copies (Berezikov, 2011).

There is in contrast a high variability in strand preference among *miR-9* copies (see Figure 1A). Upon association of microRNA duplexes with the RISC complex, only one strand is retained while the other is discarded. For most microRNAs, one of the two arms, either the 5' or 3', is preferentially selected at this step (sometimes called guide strand), while the other tends to be used more infrequently (passenger strand or star strand). In the case of *miR-9* genes, the guide strand can be generated either from the 5' (miR-9-5p) or the 3' arm (miR-9-3p) depending on the gene considered. In deuterostomes, *miR-9* genes always show a preferential usage of the 5' strand (miR-9-5p), although the 3' strand (miR-9-3p) is still present at detectable levels. This explains why miR-9-5p is often referred to as miR-9, while miR-9-3p is referred to as miR-9*. In *Drosophila* and nematode the strand bias is different for the different copies (Lim et al., 2003; Lai et al., 2004). For instance, for 3 of the 5 fly genes (*miR-9*a, *miR-9*b and *miR-9*c) the preferred strand is the 5' strand, while it is the 3' strand in the other two genes (*miR-4* and *miR-79*) (Lai et al., 2004). The bias in strand usage in this species is incidentally reflected in the gene nomenclature. Yet a different situation is observed in the marine snail *Aplysia californica*. In this species of mollusk there is only one *miR-9* gene with no preferential strand usage, mature microRNAs being equally recovered from both 5' and 3' strands of the duplex (Rajasethupathy et al., 2009). Altogether these data show that strand preference in *miR-9* genes has been quite labile during the course of evolution which certainly influenced the regulation and functional evolution of the gene family.

### Functional evolution of miR-9: implication of miR-9a in fly neurogenesis

Large scale analysis of microRNAs expression revealed that miR-9 is highly enriched in both the developing and mature nervous system of vertebrates (Miska et al., 2004; Sempere et al., 2004; Wienholds et al., 2005; Heimberg et al., 2010). Functional analyses in vertebrate model species have highlighted a prominent role of miR-9 in regulating the behavior of neural progenitors, as well as the differentiation of some neuronal populations (see further sections). The expression of miR-9/9* in human fibroblasts, in synergy with miR-124, is sufficient to convert them into neurons, placing miR-9/9* at the core of the gene network controlling the neural fate (Yoo et al., 2011). The presence of miR-9 in nervous cells might be an ancestral characteristic of bilaterian animals, as it has been observed in cephalochordate and annelid species (Christodoulou et al., 2010; Candiani et al., 2011).

**Figure 2:**
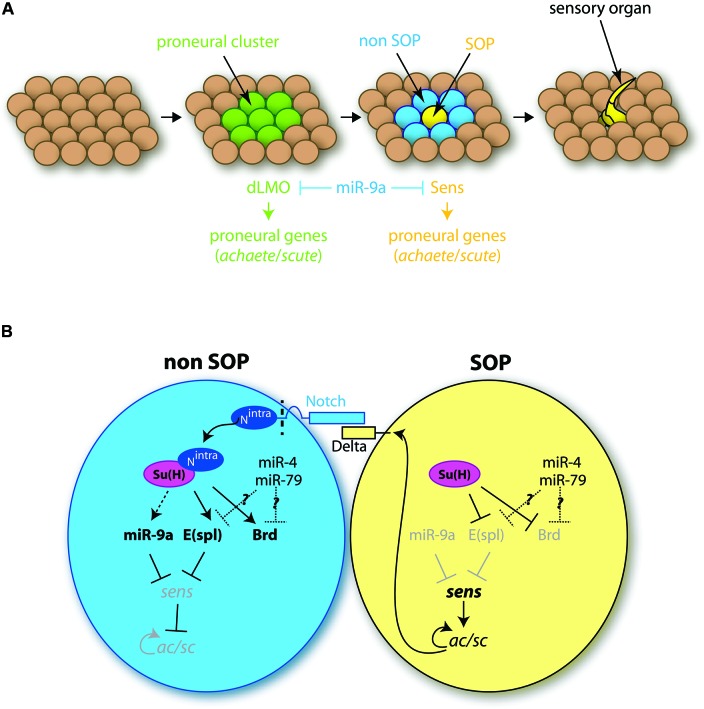
**Role of miR-9 in the development of drosophila sensory organs.** **(A)** Different steps of the formation of sensory organs. Among the ectodermal tissue, groups of cells, called proneural clusters (green), acquire neural competence via the induction of proneural genes. The dLMO protein participates in the acquisition of this competence. Among competent cells, one will maintain high levels of proneural genes expression, notably of the *sense* gene, and become a SOP cell (SOP, yellow). Concomitantly the neural fate is inhibited in the neighboring cells (non-SOP, blue). The SOP cell later divides to give rise to a sensory organ. miR-9a is present in all ectodermal cells except the SOP cell, and inhibits dLMO and Sens protein expression. **(B)** Role of miR-9a in SOP cell specification. The SOP cell expresses high levels of the ligand Delta, which interacts with Notch receptors located at the surface of neighboring cells. This interaction leads to Notch cleavage, which releases Notch intracellular domain (N^intra^) in non-SOP cells. N^intra^ interacts with the transcription factor Suppressor of Hairless Su(H), which induces *E(spl)* gene expression. *E(spl)* genes encode transcriptional repressors inhibiting the expression of proneural genes and in particular *sens*. In the SOP cell, in the absence of N^intra^, Su(H) has an inhibitory effect on the transcription of *E(spl)* genes which allows for the expression of *sens*. Sens activates the expression of proneural genes of the *ac*/*scute* complex (*ac/sc*) which specify the SOP cell fate. miR-9a, expressed in non-SOP cells, prevents ectopic expression of *sens*, thereby conferring robustness to the developmental program. Other genes of the *miR-9* family might also play a role here, as miR-4 and miR-79 have been shown to regulate the expression Notch target genes, such as *E(spl)* or *Brd*.

However, in *Drosophila*, although miR-9a does influence the development of peripheral nervous system sensory organs, its function is encoded negatively, through miR-9 restricted expression in non-neural epidermal cells (Li et al., 2006). In wild-type flies sensory precursor (SOP) cells are specified in invariable numbers among ectodermal epithelial cells in a two-step process (Skeath and Carroll, 1994; Figure 2A). First, small groups of cells, the proneural clusters, acquire neural competence via the induction of proneural genes encoding basic helix-loop-helix (bHLH) proteins such as Achaete and Scute (Ac/Sc). Among cells of the proneural cluster, as a consequence of a lateral inhibition mechanism via the Notch signaling pathway, one cell will express higher levels of proneural genes and become a SOP (Figure 2B). Mutant *miR-9*a flies display a few ectopic sensory neurons in the embryo and in the adult, a phenotype resulting from the specification of an excessive number of SOP cells (Li et al., 2006). *miR-9*a mutant phenotype is however quite mild, and its penetrance is influenced by the genetic background (Li et al., 2006; Bejarano et al., 2010). miR-9a is therefore not a strong deterministic factor, but rather confers robustness to this developmental program, a role frequently undertaken by microRNAs (Ebert and Sharp, 2012). Remarkably miR-9a influences both steps of SOP specification, through the inhibition of two major targets, *drosophila LIM-only* (*dLMO*) and *senseless* (*sens*) (Figure 2A; Li et al., 2006; Biryukova et al., 2009; Bejarano et al., 2010). *dLMO* encodes a component of a multimeric transcriptional complex shown to participate in the initial induction of *ac*/*sc* expression in proneural clusters (Ramain et al., 2000; Asmar et al., 2008). Like *miR-9*a mutants, *dLMO* gain of function mutants display extra sensory bristles (Asmar et al., 2008). These *dLMO* mutants lack large portions of *dLMO* 3'UTR, which contains a miR-9a binding site, conserved among Drosophila species, and through which miR-9a was shown to directly repress the production of the dLMO protein (Biryukova et al., 2009; Bejarano et al., 2010). *sens* is first expressed in proneural cluster cells and later accumulates at high levels in the prospective SOP (Nolo et al., 2000). Sens acts as a binary switch factor: present at low levels in proneural cluster cells, it limits the expression of *ac*; in contrast, high levels of Sens activates proneural genes expression in the SOP cell, contributing to its specification (Jafar-Nejad et al., 2003). Similar to *dLMO*, *sens* 3'UTR harbors miR-9a putative binding sites and mediates an interaction with miR-9a, which impacts on Sens protein levels (Li et al., 2006). Heterozygosity of either *dLMO* or *sens* can partially rescue the excessive number of bristles in *miR-9*a mutants, demonstrating that de-repression of these targets contributes to this phenotype (Bejarano et al., 2010). Little is known however about the upstream regulators of *miR-9*a in flies and how its expression becomes restricted to non-neural cells. The only known miR-9a regulator is the RNA-binding protein transactive response DNA-binding protein 43 (TDP-43), which seems to stabilize miR-9a pri-miR (Li et al., 2013). However, the function of other *miR-9* genes, and in particular of *miR-4* and *miR-79*, which use the opposite strand, has yet to be directly addressed in drosophila. Both miR-4 and miR-79 have been shown to interact with Bearded (Brd) box, a sequence motif previously identified as enriched on the 3'UTR of Notch target genes such as *Enhancer-of-split* (*E(spl)*) or *Brd* (Lai and Posakony, 1997; Lai et al., 2005; Figure 2B). One could thus hypothesize a role of these micro­RNAs in neurogenesis through the deregulation of Notch target genes.

In sharp contrast with the situation in vertebrates, studies in drosophila thus point to an anti-neural role of miR-9a: expressed in non-neural epithelial cells, miR-9a restricts the number of specified SOP by dampening the expression of the proneural genes *dLMO* and *sens*. This suggests that, despite the fact that miR-9a sequence is strictly identical to its vertebrate counterparts, its function and its set of targets have been profoundly remodeled during evolution.

## miR-9 regulation of neural development

In deuterostomes, *miR-9* genes demonstrate a strong evolutionary plasticity, in terms of strand usage and developmental function. In contrast, studies in vertebrate model species point to highly conserved functions of miR-9, especially in the regulation of neural progenitor proliferation.

### miR-9 regulation of neural progenitors

#### miR-9 expression is preferentially associated with neurogenic progenitors

**Figure 3:**
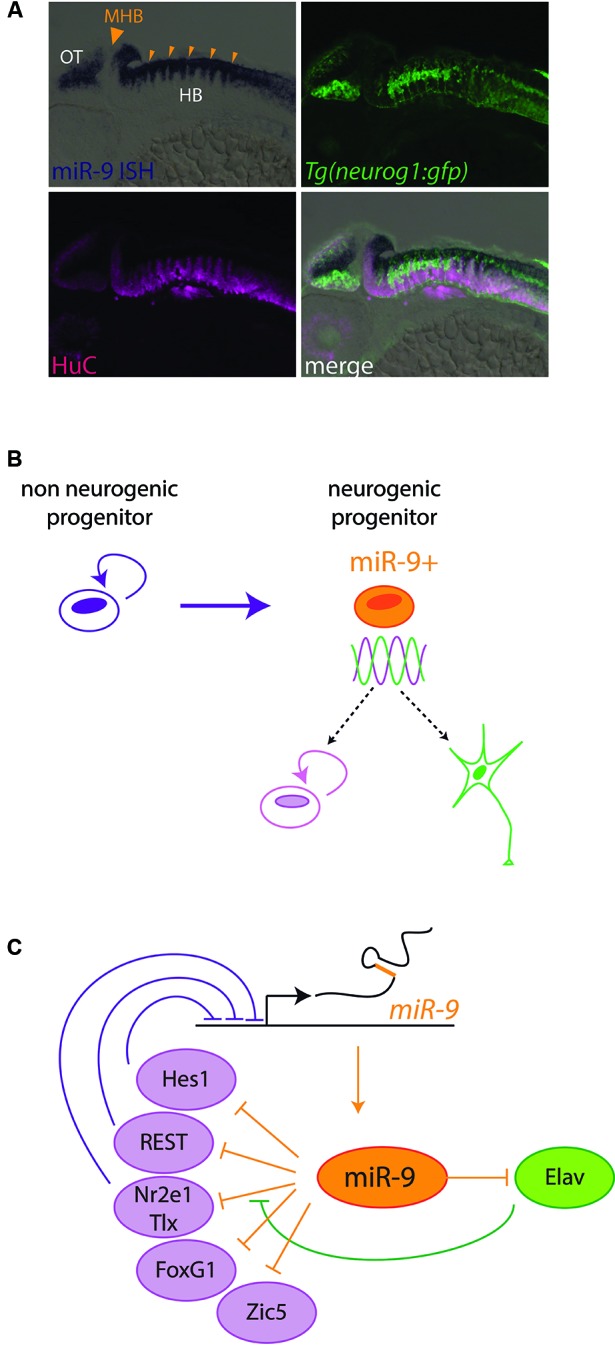
**miR-9 regulates progenitor states in Vertebrates.** **(A)** miR-9 is expressed in active neurogenic zones. Sagittal section through a zebrafish embryo at 48 h post fertilization, showing the expression of miR-9 as revealed by *in situ* hybridization (blue). miR-9 is expressed at the ventricular zone, and excluded from differentiated neurons expressing the protein HuC (magenta). Its expression is induced in neurogenic areas, where the expression of proneural genes such as *neurogenin1* (*neurog1*) is detected (green). In contrast, miR-9 is excluded specifically from boundary regions, containing long-lasting neural progenitors, such as the MHB (big arrowhead) or rhombomere boundaries (small arrowheads). **(B)** Functional data suggest that miR-9 promotes the transition from a non-neurogenic progenitor, expressing high levels of Hes1, to a neurogenic progenitor, in which Hes1 levels oscillate. The miR-9 expressing neurogenic progenitor is in an ambivalent state, poised to respond to proliferation or differentiation cues. **(C)** Scheme representing negative feedback loops between miR-9 and its targets, some of which promoting proliferation (purple) and others promoting differentiation (green).

The first large scale microRNA expression profiles performed in vertebrates soon identified miR-9 as a brain enriched microRNA (Lagos-Quintana et al., 2002; Krichevsky et al., 2003; Miska et al., 2004; Sempere et al., 2004). In particular, its expression levels were shown to be dynamically regulated during brain development, and during *in vitro* induced neurogenesis (Miska et al., 2004; Sempere et al., 2004; Krichevsky et al., 2006). miR-9*, derived from the 3' strand of *miR-9* genes, is also present at detectable levels in vertebrate neural tissues. *In situ* hybridization analyses in different vertebrate model organisms have revealed very similar spatiotemporal patterns of miR-9 expression during (CNS) development (Darnell et al., 2006; Deo et al., 2006; Leucht et al., 2008; Shibata et al., 2008; Walker and Harland, 2008). miR-9 expression starts at mid-embryogenesis stages, after the specification of the major brain subdivisions and the development of the primary neuronal scaffold. Its expression is first induced in the telencephalon, and later spreads to more posterior brain regions and spinal cord. All along the CNS, miR-9 expression is predominantly associated with ventricular neural progenitors areas (Darnell et al., 2006; Leucht et al., 2008; Shibata et al., 2008, 2011; Bonev et al., 2011; Coolen et al., 2012), although some differentiated neurons also express miR-9, notably in the dorsal telencephalon and spinal cord (Leucht et al., 2008; Otaegi et al., 2011; Shibata et al., 2011). Its expression characterizes active neurogenic areas and its expression is dependent on the activity of Notch signaling (Coolen et al., 2012). In contrast, miR-9 seems to be specifically excluded from progenitor pools located at boundaries between brain compartments, such as the Midbrain-Hindbrain Boundary (MHB) or rhombomeres boundaries (Figure 3A; Leucht et al., 2008; Coolen et al., 2012). In these boundary regions, which play a role as late signaling centers, neural progenitors do not enter neurogenesis and remain undifferentiated over long periods (Kiecker and Lumsden, 2005). Interestingly, miR-9 expression appears similarly regulated in the retina. There miR-9 expression is also restricted to late progenitors (La Torre et al., 2013) and shows a dependence on Notch activity (Georgi and Reh, 2010). Furthermore it appears to be excluded from the progenitor pool located at the ciliary marginal zone in non-mammalian vertebrates (Kapsimali et al., 2007). This pool maintains undifferentiated progenitors over a long time, reminiscent of neural tube boundary regions. miR-9 was also detected in adult brain neurogenic areas, in both fish and mouse (Deo et al., 2006; Kapsimali et al., 2007; Tozzini et al., 2012), and in primary cultures of mouse adult stem cells (Zhao et al., 2009). However a precise characterization of miR-9-positive cells in adult brains remains to be performed.

#### Regulation of neural progenitors proliferation by miR-9

As the expression of miR-9 suggests, functional analyses uncovered a prominent role of miR-9 in the regulation of embryonic neural progenitors states. In zebrafish embryos, miR-9 was shown to participate in the late patterning of the midbrain/hindbrain region. The activity of miR-9 on both sides of the MHB restricts the extent of the pool of non-neurogenic progenitors located at this boundary (Leucht et al., 2008). However, miR-9 has a more general influence on the behavior of neurogenic progenitors along the neural tube. Overexpression of miR-9/9* duplexes in the zebrafish embryo (Leucht et al., 2008), mouse embryonic cortex (Zhao et al., 2009), and chick spinal cord (Yoo et al., 2011) leads to a reduction in the number of proliferating progenitors. This effect is accompanied by precocious neuronal differentiation (Leucht et al., 2008; Zhao et al., 2009). miR-9/9* was also shown to promote differentiation of adult neural stem cells *in vitro*, albeit only if they were primed for differentiation beforehand using forskolin or retinoic acid (Zhao et al., 2009). Additionally, infecting human neonatal fibroblasts with lentiviral vectors containing miR-9/9* and miR-124 induces their conversion into postmitotic neurons. However, this conversion is dependent on the expression on all three microRNAs (Yoo et al., 2011). Thus, these *in vitro* data show that miR-9 alone is not sufficient to induce neuronal differentiation. Conversely, miR-9 loss-of-function consistently induces an increased proliferation of embryonic neural progenitors (Bonev et al., 2011; Shibata et al., 2011; Coolen et al., 2012) or mouse adult neural stem cells (Zhao et al., 2009). However neural progenitors resume differentiation even under miR-9 depletion conditions, their cell cycle exit being only delayed (Shibata et al., 2011; Coolen et al., 2012). Altogether this data suggests that miR-9 does not act as a necessary and sufficient differentiation switch, but rather could favor the transition of progenitors from a proliferative mode to a neurogenic mode. Of note, in over-expression experiments or in mouse mutants, the effects observed result from the combined gain or loss of both miR-9 strands. In contrast, in depletion experiments using antisense oligonucleotides, like the one performed in zebrafish or Xenopus embros, only miR-9 (miR-9-5p) is down-regulated. The individual role of the other miR-9* (miR-9-3p) has yet to be assessed. Moreover, in human embryonic stem cell-derived neural progenitors, and rat embryonic cortical progenitors, miR-9 was shown to have a completely opposite role (Delaloy et al., 2010). In this study, inhibition of miR-9 led to a decrease in neural progenitor proliferation, concomitant with increased migratory capacities. The different effects observed on neural progenitors in culture might be linked to a different timing of miR-9 depletion during *in vitro* differentiation (Zhao et al., 2009; Delaloy et al., 2010). These results indicate that miR-9 can inversely impact the proliferation of neural progenitors depending on the cellular context. Differential expression of mRNA targets or the synergy between miR-9 and other mRNA regulating factors could account for this phenomenon. For instance the RNA binding proteins Elavl1 and Musashi1 can synergize with miR-9 to increase the expression of some of its targets (Shibata et al., 2011). A better appreciation of the spatial and temporal variations of miR-9 function *in vivo* awaits the development of more refined conditional knock-down tools.

#### miR-9 targets in neural progenitors reveal a complex interacting network

A better understanding of miR-9 function will certainly arise from the characterization of its set of targets and the analysis of the impact of these interactions *in vivo*. *In silico* algorithms predict several hundred targets for miR-9, a high figure typical of ancient microRNAs (Bartel, 2009). So far, only a few of these interactions have been confirmed *in vitro* or *in vivo*, but these studies have shed light on the complex mode of action of miR-9 in neural progenitors.

A first set of miR-9 targets are members of the *Hes* gene family (Leucht et al., 2008; Bonev et al., 2011, 2012; Coolen et al., 2012). *Hes* genes are the main Notch signaling effectors and encode transcriptional repressors (Kageyama et al., 2008). Expressed in neural progenitors, they inhibit differentiation by repressing proneural genes such as *ascl1*, a vertebrate homolog of *Drosophila* *ac*/*sc* complex genes. Some of them are also highly expressed at boundaries and help in maintaining slow cycling non-neurogenic progenitors at these locations (Kageyama et al., 2008; Stigloher et al., 2008). Among miR-9 targets is *her5*, which in zebrafish embryos is specifically expressed at the MHB. In this area miR-9 restricts the size of the MHB progenitor pool through repressing *her5* while simultaneously limiting the signaling activity of the MHB progenitors by repressing Fibroblast growth factor (FGF) pathway genes (Leucht et al., 2008). *Hes1/her6* genes also harbor a miR-9 binding site in their 3'UTR, which is conserved across vertebrates. In zebrafish and frog embryos, target protector morpholinos (Choi et al., 2007) that block the miR-9 binding site on the *hes1*/*her6* 3'UTR, induce an increased proliferation, mimicking the effect of miR-9 blockade (Bonev et al., 2011; Coolen et al., 2012) and thus demonstrate that miR-9 targeting of this gene is crucial to properly balance progenitor proliferation. Interestingly, *Hes1* was previously shown to be expressed in neural progenitors both at boundary regions and inside brain compartments. However, live imaging using a *Hes1* luciferase reporter demonstrated that at boundaries, *Hes1* expression is high and stable, whereas it displays ultradian oscillations inside brain compartments (Shimojo et al., 2008). This difference in *Hes1* expression mode is thought to parallel the non-neurogenic versus neurogenic properties of these two kinds of progenitors (Kageyama et al., 2008). In an *in vitro* model, the dampening of *Hes1* expression by miR-9 was shown to be necessary for oscillations of *Hes1* to occur (Bonev et al., 2012; Tan et al., 2012). miR-9 could therefore promote a transition towards neurogenesis via influencing the mode of *Hes1* expression.

Several other validated targets of miR-9 are also transcription factors that were previously shown to promote progenitor proliferation. They include notably the forkhead transcription factor FoxG1 (Shibata et al., 2008, 2011), the homeobox factor Gsx2 (Shibata et al., 2011), the orphan nuclear receptor Tlx/Nr2e1 (Biryukova et al., 2009; Bonev et al., 2011) and the zinc finger transcription factor Zic5 (Coolen et al., 2012). The repression of these transcription factors by miR-9 could explain the anti-proliferative effect it exerts in neural progenitors, while their up-regulation could participate in miR-9 the depletion phenotype. Thus in *miR-9*-2/3 double mutant mice, the clear increase in FoxG1 and Gsx2 protein levels could contribute to increasing proliferation of embryonic pallial and subpallial progenitor cells (Shibata et al., 2011). Moreover, the reduction of proliferation induced by miR-9 can be rescued by overexpressing TLX, suggesting that the inhibition of this target could participate in this phenotype (Zhao et al., 2009). Surprisingly however, the levels of TLX proteins are not up-regulated but down-regulated in *miR-9*-2/3 double mutant mice (Shibata et al., 2011). This discrepancy could be linked to the presence of the RNA binding proteins Elavl1 or Msi1, which were shown *in vitro* to be able to convert miR-9 to an activator (Shibata et al., 2011). Alternatively, indirect effects of miR-9 depletion could also explain the reduction of TLX protein. Zic5 belongs to a family of Zinc finger transcription factors that act as inhibitors of neuronal differentiation during development (Aruga, 2004). In vertebrates, *Zic5* mRNA possesses a very conserved binding site for miR-9, and moreover, in the zebrafish embryonic hindbrain, injection of a target protector morpholino restricting binding to this site leads to an increase in progenitor proliferation (Coolen et al., 2012).

Other targets of miR-9 are linked with the epigenetic machinery, which is subjected to drastic remodeling during the course of neuronal differentiation. The 3'UTR of repressor-element-1 silencing transcription factor (REST) and the corepressor CoREST, were shown to harbor functional binding sites for miR-9 and miR-9* respectively (Packer et al., 2008). REST and CoREST act in a chromatin-bound protein complex, which recruits histone modifiers to repress the expression of neuronal genes in neural stem cells and progenitors (Ballas and Mandel, 2005). During neuronal differentiation, the complex is dismantled, which allows for the expression of neuronal genes. During the transition from progenitors to neurons, there is also an exchange of subunits within the Switch/Sucrose non fermentable (Swi/Snf) chromatin remodeling complex: the subunit BRG1- and BRM-associated factor 53a (BAF53a) present in neural progenitors is notably replaced by its homologous BAF53b. Interestingly, miR-9* seems to be able to facilitate this exchange, via repressing the expression of BAF53a (Yoo et al., 2009). miR-9 can also inhibit the expression of Sirt1, a member of the class III nicotinamide adenine dinucleotide (NAD+)-dependent histone deacetylases (Delaloy et al., 2010). Sirt1 associates with different repressor complexes and opposite influences of this factor have been observed during *in vitro* neurogenesis: Sirt1 can associate with Hes1 to repress proneural gene expression (Prozorovski et al., 2008), however, on the other hand, Sirt1 translocation to the nucleus was also shown to accompany neuronal differentiation of neural stem cells *in vitro* and accelerate this process via repressing Notch targets such as Hes1 (Hisahara et al., 2008). It is therefore hard to draw a conclusion on the potential functional consequences of miR-9 repression of Sirt1 at this stage. Importantly the significance of these microRNA-targets interactions remains to be directly assessed *in vivo*. Nevertheless they suggest exciting links between miR-9 and the epigenetic landscape of neural progenitor cells. Interestingly miR-9 could also participate in remodeling the microRNAs landscape in neural cells. Indeed miR-9 can inhibit the pluripotent factors Lin28A and Lin28B, RNA binding proteins that block the processing of some microRNAs, including let-7 (Eda et al., 2009; La Torre et al., 2013).

miR-9 can promote neural differentiation via the inhibition of proliferation factors and progenitor specific epigenetic factors. Surprisingly however, miR-9 was shown to also downregulate the expression of genes with differentiation promoting activities. For instance, using target protector morpholinos, a cryptic role of miR-9 in inhibiting *elavl3*, a proneural differentiation factor, was revealed in zebrafish embryos (Coolen et al., 2012). In doing this, miR-9 dampens the expression of factors favoring antagonist fates. Thereby it seems to favor an ambivalent progenitor state, poised to respond to both progenitor maintenance and commitment cues (Figure 3B). This ambivalent state could possibly correspond to the previously described “oscillating” neurogenic progenitors in which the levels of opposite fate determinants like Hes1 and Ngn2 oscillate prior to commitment (Kageyama et al., 2008). Another striking feature emerging from the analyses of miR-9 targets is that they often exert feedback regulation on miR-9 (Figure 3C). Transcription of *miR-9* genes is repressed by Nr2e2/Tlx, Hes1 and REST, and its effect on mRNA targets can be modulated by Elavl proteins, also targeted by this microRNA (Zhao et al., 2009; Laneve et al., 2010; Shibata et al., 2011; Bonev et al., 2012). Feedback loops are recurring motifs in gene regulatory networks involving microRNAs, and they can stabilize cellular states and provide robustness to developmental programs (Peláez and Carthew, 2012). Altogether the study of miR-9 targets point to a role of this microRNA to facilitate, pace and stabilize the transition of progenitors towards neural differentiation. To obtain a full picture of its gene network a thorough characterization of miR-9 targets and validation of the impact of individual interactions should be conducted. This would help understanding the phenotype resulting from its absence, which combines the sometimes opposite effects of the deregulation of many mRNAs.

### Additional roles of miR-9 in neural development: miR-9 in post-mitotic neurons

A few studies have unraveled additional functions of miR-9 at later steps of neural development, linked to its expression in some populations of post-mitotic neurons.

miR-9 is transiently expressed during the differentiation of spinal cord motoneurons (MN), located in the lateral motor column and innervating limb muscles, but not in neighboring MN (Otaegi et al., 2011). These MN are characterized by the expression of FoxP1 and Isl1/2, both of which are putative targets of miR-9. In this context, manipulating miR-9 levels impairs the differentiation and axonal projections of spinal motoneuron lineages, possibly through a de-regulation of FoxP1 protein levels.

The paralog gene *Foxp2* also possesses functional miR-9 binding sites on its 3'UTR. Foxp2 protein is expressed in the embryonic cortex, but its expression starts much later than miR-9. Furthermore, while miR-9 expression spans most layers of the cortex, being however enriched in ventricular progenitors, Foxp2 expression is restricted to post-migratory neurons (Shibata et al., 2011; Clovis et al., 2012). Both miR-9 putative binding sites can mediate the repression of a reporter transgene by endogenous miR-9 present in the cortex (Clovis et al., 2012). This suggests that miR-9 could prevent excessive expression of the protein FoxP2 in the cortex, which was shown to severely impair neuronal migration. Moreover excessive expression of FoxP2 also compromises maturation of cortical neurons.

A role of miR-9 in the maturation of cortical neurons was more directly demonstrated by another study in which miR-9 expression could be detected in axons and dendrites of differentiated neurons (Dajas-Bailador et al., 2012). Moreover, inhibition of miR-9 in cultured neurons increased axonal length and reduced axonal branching. Specific inhibition of miR-9 binding on the *Map1b* 3'UTR both *in vitro* and *in vivo* using target protectors could mimic this effect of miR-9. Thus miR-9 regulates neuronal maturation through modulating the expression level of this gene, which is an important regulator of microtubules dynamics.

## Implication of miR-9 in human pathologies

### miR-9 in cancer

Studies in the developing brain demonstrated that miR-9 is deeply rooted in gene networks controlling the regulation of neural progenitors proliferation. It is therefore not surprising to see this microRNA implicated in the progression of brain cancers such as medulloblastoma and glioblastoma. As in the normal contexts, this microRNA demonstrates its versatility in the context of tumors.

Medulloblastomas (MB) are the most frequent form of pediatric brain cancers. They originate from cerebellar progenitors. miR-9 expression is reduced in MB samples compared to neighboring brain tissues (Ferretti et al., 2009). This could contribute to disease progression as inhibition of miR-9 in MB cell lines increases their proliferation. One target of miR-9, the truncated form of the neurotrophin receptor TrkC (t-TrkC), is up-regulated in MB and was shown to promote proliferation of MB cells. The deregulation of t-TrkC following down-regulation of miR-9 could therefore play a role in sustaining proliferation of MB cells. Conversely a high expression of miR-9 was detected in a subclass of glioblastoma, the most common but also the most aggressive type of adult brain tumors (Kim et al., 2011). Interestingly, miR-9 expression was particularly associated with tumor cells possessing stem-like features (Schraivogel et al., 2011). These cells, referred to as glioblastoma stem cells (GSC), are defined by long term self-renewal capacities thus endowing them with greater potential for cancer initiation and propagation (Huang et al., 2010). Several studies demonstrate that these cells are particularly resistant to radiotherapy and chemotherapy, and are therefore likely responsible for tumor resistance and recurrence. Reducing miR-9 expression in glioblastoma primary culture leads to a reduction of the number of cells with *in vitro* self-renewing potential (Schraivogel et al., 2011). This effect is mediated by an up-regulation of calmodulin-binding transcription activator 1 (CAMTA1), a tumor suppressor whose 3'UTR is targeted by miR-9. However another study suggests that miR-9* inhibits the expression of Sox2, a factor which, in contrast to CAMTA1, confers self-renewal properties and drug resistance to GSC (Jeon et al., 2011). miR-9 impact on GSCs and tumor growth seems therefore to be variable among glioblastoma samples, an observation likely to reflect the high heterogeneity of these tumors. It would however be particularly interesting to investigate further the link between miR-9 and GSCs properties and compare miR-9 action in normal versus tumoral stem cells.

Surprisingly, miR-9 was also linked with cancers originating outside the nervous system. In some cases, miR-9 behaves like an oncogene, in some others like a tumor suppressor. miR-9 is highly expressed and appears to favor progression of Hodgkin lymphomas (Leucci et al., 2012), breast cancers (Ma et al., 2010), cervical cancers (Wilting et al., 2013), colon cancers (Lu et al., 2012) and stomach cancers (Rotkrua et al., 2011). The proximal causes of miR-9 up-regulation have been identified only in a few cases. Chromosomal amplifications can account for increased miR-9 expression in some cervical cancers (Wilting et al., 2013), while the up-regulation of *miR-9*-3 transcription is caused by the MYC oncogene in some breast cancers (Ma et al., 2010). miR-9 has been shown to influence various tumorigenic processes, including cellular proliferation (Rotkrua et al., 2011; Wilting et al., 2013), migration (Ma et al., 2010; Lu et al., 2012) and inflammation (Leucci et al., 2012). A reduction of miR-9 expression compared to normal tissue was also observed in other types of cancer, including leukemia (Senyuk et al., 2013), lung cancers (Heller et al., 2012) and colon cancers (Bandres et al., 2009). The implication of miR-9 in tumorigenesis in such a variety of tissues suggests that miR-9 may regulate general processes and define a specific cellular state that could exist outside the nervous system.

### miR-9 in neurodegenerative disorders

Links between neurodegenerative disorders and microRNAs, especially brain enriched microRNAs such as miR-9, have started to emerge in the literature (Lau and de Strooper, 2010). Huntington’s disease (HD) is an autosomal dominant neurodegenerative disease caused by trinucleotide repeat expansions in the *Huntingtin* gene (*Htt*). One hallmark of HD is an alteration of the transcriptome of some brain regions, linked to abnormal activity of the transcriptional repressor REST (Buckley et al., 2010). In contrast to the wild-type HTT, mutant HTT no longer traps REST in the cytoplasm, thus allowing it to translocate to the nucleus and excessively represses its targets. As previously mentioned, REST was shown to repress *miR-9* transcription and REST and its co-repressor coREST are targets of miR-9/9* (Packer et al., 2008; Laneve et al., 2010). In the brain of HD patients, miR-9/9* expression decreases with increasing disease grades (Packer et al., 2008). The down-regulation of miR-9/9* could be linked to the excessive activity of REST and may also amplify REST activity by increasing REST and coREST protein levels. Considering the number of targets of miR-9/9*, their down-regulation could also impact more widely the transcriptional landscape in HD patients brains. miR-9 was also found to be deregulated in other neurodegenerative disorders. It is down-regulated in the brain of Alzheimer’s disease patients (Cogswell et al., 2008; Hébert et al., 2008) and up-regulated in the cortex of Parkinson’s disease patients (Kim et al., 2007). Finally, miR-9 was suggested to participate in amyotrophic lateral sclerosis (ALS), a neurodegenerative disease affecting MN (Haramati et al., 2010). miR-9 was shown to be down-regulated in a mouse model of motoneuron disease and its over-expression can repress the expression of a neurofilament heavy subunit previously linked to motoneuron degeneration. Probing more directly the impact of miR-9 deregulation on neurodegenerative disease progression is a challenging task, which will need to be addressed in the future.

### miR-9 protects neural tissue from deleterious Progerin

**Figure 4:**
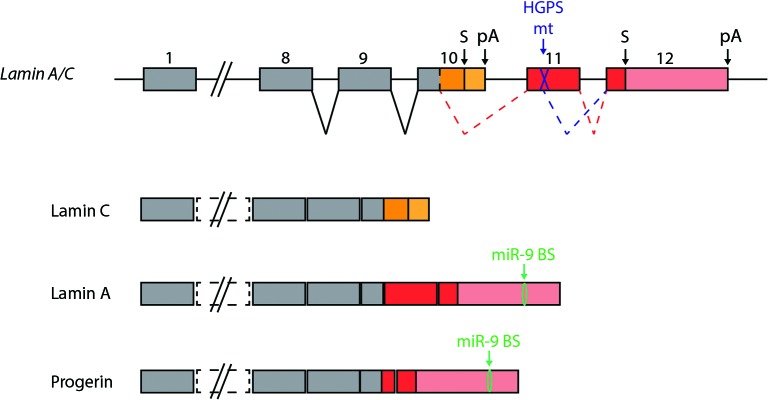
**miR-9 protects the brain from Progerin.** Two alternative transcripts are generated from the the Lamin A/C gene, encoding Lamin C and Lamin A proteins. HGPS is caused by a mutation in exon 11, which is specific to Lamin A encoding transcripts. The mutation generates an additional splice site, which leads to the generation of a new transcript encoding a truncated form of Lamin A. The truncated protein is referred to as Progerin, and is toxic to cells. The presence of miR-9 in the central nervous system can explain, at least in part, the low levels of Lamin A detected in this tissue compared to Lamin C. In HGPS patients, miR-9 repression of Progerin expression protects the CNS from this harmful protein.

Hutchinson-Gilford progeria syndrome (HGPS) is a rare disease whose symptoms resemble physiological ageing. It is caused by a de novo specific mutation in *LMNA* gene. This gene encodes two components of the nuclear lamina, lamin A and lamin C, as a result of alternative splicing (Figure 4). HGPS causing mutations affect only lamin A-encoding transcripts and generate a truncated form of this protein which is toxic to cells, referred to as Progerin. While most tissues and organs are affected by the presence of Progerin, the CNS of HGPS-affected patients is remarkably spared. This is most likely due to a lower expression of lamin A in neural cells, but the underlying mechanism remained unknown. Two recent studies demonstrated a role of miR-9 in this process (Jung et al., 2012; Nissan et al., 2012). The first study confirmed that lamin A transcripts and proteins are less abundant than lamin C in mouse brains, whereas they are expressed at similar levels in other tissues (Jung et al., 2012). Low levels of Progerin were also found in the brain of knock-in mice harboring the HGPS mutant allele. The authors could show that the differential levels of lamin A versus lamin C in brain tissues was not linked to differences in alternative splicing, but rather that it is likely linked to the presence of miR-9 in neural tissues. Lamin A and C encoding transcripts possess different 3'UTR sequences, but only lamin A transcripts can be down-regulated by miR-9 through a functional binding site. Inhibition of miR-9 in neural cells *in vitro* lead to increased levels of lamin A transcripts. These results were confirmed to be relevant in human patients by the second study, in which neural cells differentiated from HGPS patient-induced pluripotent stem cells (iPSC) were analyzed (Nissan et al., 2012). Neural stem cells and neurons derived from patient iPSCs express lower levels of lamin A and Progerin transcripts compared to other iPSC derived cell types. These cells are also characterized by high levels of miR-9. Over-expression of miR-9 in mesenchymal cells of HGPS patients could reduce the levels of lamin A and progerin, an effect mediated by the 3'UTR of the lamin A transcripts. Altogether this suggests that miR-9 expression may account for the low expression of lamin A in the neural tissue, thus protecting it from the deleterious effects of Progerin in HGPS patients. This regulation could stimulate the development of new therapies for HGPS patients. The reason why lamin A expression would need to be restricted by miR-9 in a healthy brain remains to be discovered.

## Conclusion

miR-9 is a very ancient microRNA and its function and set of targets seem to have undergone dramatic changes during the evolution of Bilateria. In drosophila, through its expression in non-neural cells miR-9a confers robustness to the developmental program of peripheral organs. In contrast, in vertebrates, miR-9 displays a conserved expression pattern in the CNS. During vertebrate evolution, miR-9 seemingly had the time to accumulate a large set of mRNA targets, and is deeply embedded in the gene network controlling the behavior of neural progenitors. Strikingly, functional studies of miR-9 point to a highly versatile action. Conditional depletion of miR-9 *in vivo*, using refined genetic tools, will help better characterizing this phenomenon *in vivo*. In addition, to better understand the full repertoire of miR-9 actions, it will be necessary to identify the set of miR-9 targets in different cellular contexts and evaluate the functional impact of these interactions individually. This will constitute a major challenge, especially since miR-9 targets are functionally interconnected. For most targets studied so far in vertebrates, miR-9 binding sites are highly conserved and are thus part of an ancestral set of miR-9 targets. It would be interesting now to start to explore species-specific targets of miR-9 and see how they could participate in the diversification of the nervous system. Finally, to investigate the variability of miR-9 actions, it will be of interest to identify its regulators. In vertebrates, the transcription factors that were shown to bind *miR-9* promoters are all repressors (Tlx, REST, Hes1); the factors that can induce miR-9 expression are still unknown. Further studying the link between miR-9 activity and RNA binding proteins, which can either regulate miR-9 expression and processing or alter its effect of mRNA targets, will refine our understanding of miR-9 action (Shibata et al., 2011; Xu et al., 2011; Li et al., 2013). Altogether, a comprehensive knowledge of how miR-9 function can be modulated across different species and cellular contexts will be necessary to unravel its contribution in human pathologies.

## Conflict of interest statement

The authors declare that their research was conducted in the absence of any commercial or financial relationships that could be construed as a potential conflict of interest.

## Footnotes

^1^http://www.mirbase.org
